# Advanced FFF of PEEK: Infill Strategies and Material Characteristics for Rapid Tooling

**DOI:** 10.3390/polym15214293

**Published:** 2023-11-01

**Authors:** Karim Abbas, Lukas Hedwig, Nicolae Balc, Sebastian Bremen

**Affiliations:** 1 Department of Mechanical Engineering, University of Applied Sciences Aachen, 52064 Aachen, Germany; hedwig@fh-aachen.de (L.H.); bremen@fh-aachen.de (S.B.); 2 Department of Manufacturing Engineering, Technical University of Cluj-Napoca, 400641 Cluj-Napoca, Romania; nicolae.balc@tcm.utcluj.ro

**Keywords:** additive manufacturing, fused filament fabrication, compression behavior, infill strategy, rapid tooling, polyetheretherketone (PEEK)

## Abstract

Traditional vulcanization mold manufacturing is complex, costly, and under pressure due to shorter product lifecycles and diverse variations. Additive manufacturing using Fused Filament Fabrication and high-performance polymers like PEEK offer a promising future in this industry. This study assesses the compressive strength of various infill structures (honeycomb, grid, triangle, cubic, and gyroid) when considering two distinct build directions (Z, XY) to enhance PEEK’s economic and resource efficiency in rapid tooling. A comparison with PETG samples shows the behavior of the infill strategies. Additionally, a proof of concept illustrates the application of a PEEK mold in vulcanization. A peak compressive strength of 135.6 MPa was attained in specimens that were 100% solid and subjected to thermal post-treatment. This corresponds to a 20% strength improvement in the Z direction. In terms of time and mechanical properties, the anisotropic grid and isotropic cubic infill have emerged for use in rapid tooling. Furthermore, the study highlights that reducing the layer thickness from 0.15 mm to 0.1 mm can result in a 15% strength increase. The study unveils the successful utilization of a room-temperature FFF-printed PEEK mold in vulcanization injection molding. The parameters and infill strategies identified in this research enable the resource-efficient FFF printing of PEEK without compromising its strength properties. Using PEEK in rapid tooling allows a cost reduction of up to 70% in tool production.

## 1. Introduction

The production of injection molds is a complex manufacturing process involving many manufacturing steps and iterative development processes. Rapid tooling is an essential field of application for additive manufacturing [1]. Here, injection molds are produced directly using polymer or metal-based AM processes. These are suitable for prototype production as well as series production [2,3]. The field of rapid tooling is gaining prominence due to its economic efficiency and its distinct advantage over traditional manufacturing processes. In their research, Kuo et al. discovered that employing AL-filled resin in rapid tooling can lead to reductions of over 30% in production costs and manufacturing time. However, their study also revealed that the lifespan of an RT tool is merely 1.3% of that of a metal tool [4]. Similarly, Kampker et al. identified potential cost savings of up to 72% and estimated overall savings ranging from 20% to 66% [5]. A study conducted by the Swiss company LIM Technics OOD and Apium evaluated potential cost reductions of up to 86% and a time-saving of 66% using rapid tooling [6].

In polymer AM, stereolithography-based processes, in particular, have become established. A little-known process in this field is Fused Filament Fabrication (FFF for short). FFF is based on the extrusion of thermoplastics. Here, a thermoplastic is temporarily melted in a heated nozzle and then applied layer by layer. It is one of the most critical and accessible AM processes. However, due to the limited surface quality, it is only suitable for rapid tooling to a limited extent. 

The possibility of processing high-performance polymers such as polyetheretherketone using FFF opens up new possibilities in rapid tooling [7,8,9], among other things, in the production of injection molds for thermoplastics [6,10,11,12] and vulcanization molds [13] for rubber components, as an already conducted study by the authors shows [14]. 

Although using high-performance polymers can reduce costs in mold manufacturing, it is also necessary to minimize the use of resources. This should ensure companies’ competitiveness and economic viability and promote the sustainable use of polymers. At the same time, the material savings must not compromise the mechanical properties, as injection molds are subject to high mold forces and injection pressures.

The Fused Filament Fabrication process (FFF for short) is particularly well-suited for lightweight construction applications and material conservation due to its ability to adjust the component density. It distinguishes between two key aspects: perimeters and infill. Perimeters constitute the outer walls of the part, with thickness determined by specified parameters. Infill, on the other hand, represents the internal structure of the part and is defined by two primary parameters: infill density, which determines the amount of material inside the part, and infill pattern. A part with 0% infill density is hollow, while a part with 100% is filled with material [3].

The infill pattern, also called infill design, is important for the part’s strength. A widely used infill pattern is the grid or lattice pattern. It is composed of lines that are rotated 90° to each other. Another anisotropic infill is the honeycomb pattern. The bionic structure is characterized by a high strength-to-weight ratio [15]. The high strength-to-weight ratio of honeycomb structures are already being used in applications such as aerospace, automotive, medicine, and architecture. As the strength in the direction of the honeycomb is particularly high, sandwich structures are often used in which honeycombs serve as the core material [16]. In contrast to grid, triangle, and cubic infill, honeycomb infill does not consist of straight lines. During the printing process, braking and acceleration must occur at every direction change. This means that honeycomb infill requires more printing time for the same filling density. Unlike anisotropic infill, isotropic infill aims to have the same properties in all spatial directions.

For instance, the isotropic cubic infill has a cube as its unit cell. The cube is rotated in space by 45° in two planes so that the individual layers are composed of three lines rotated by 60°, like the triangle infill. In contrast to the triangle infill, the pattern is not repeated identically in each layer, and a three-dimensional structure is formed. 

The isotropic gyroid infill, however, is based on a triple periodic minimal surface, developed by Alan Schoen for NASA in 1970. This surface belongs to the family of Black P and D surfaces [17]. The name P surface is derived from the word “primitive”. The unit cell is a cube cut by a tube in the X, Y, and Z directions. All surfaces created in this way are rounded off against each other. On each outer surface of the cube, there is a circle to which the next unit cell is connected [18].

The name “Black D” surface is derived from the word “Diamond”. The surface structure is derived from a diamond structure/diamond lattice. There are also planes in the three directions rounded off about each other [18]. Both surfaces can be created mathematically by adding and multiplying sine and cosine functions [17].

The gyroid surface developed by Schoenen has neither straight nor plane curvature lines [17] to minimize load peaks [19]. Al-Ketan et al. showed for various materials that gyroid structures could withstand higher loads at the same weight as other lattice structures [20]. Abueidda et al. verified these results on polyamide samples produced with the Selective Laser Sintering process (SLS) [19]. As with the Honeycomb infill, there are many direction changes with the Gyroid infill, which increases the printing time.

Researchers investigated the effects of infill structure and density on FFF printed components’ tensile strength. Pandzic et al. [21] investigated the effect of different infill patterns on the tensile strength of PLA specimens. The characteristic values determined lie between 18.7 MPa and 35.7 MPa. The maximum values are achieved by the concentric infill, which can be explained by the orientation of the material webs in the tensile direction. High values up to 27.7 MPa are also shown by the Gyroid-Infill, which, according to the manufacturers, often indicates a higher isotropy in components. For the tensile strength, all infill patterns show an increasing strength with increasing infill density [21].

In studies on the tensile strength of PLA, Cerda et al. found that infill density and the orientation of the samples in the print space had the most significant influence on the strength of the components. More infill therefore always leads to a higher tensile strength [22].

In addition to tensile strength, the effects of FFF parameters on flexural strength have been researched. Mishra et al. investigated the flexural strength of ABS specimens. It was shown that the strength of components increases significantly when the wall thickness is increased. Components with one outer contour achieved an average of 52.3 MPa, 56.9 MPa with three contours, and 58.1 MPa with five contours [23]. According to Luzanin et al., the layer thickness significantly influences flexural strength. The interaction of orientation and infill density also considerably influenced the investigated bodies made of PLA [24].

Few studies are concerned with characterizing the compressive properties of infill patterns. Frequently, correlations between infill patterns and tensile strength are evaluated. The focus is preferably on standard and engineering polymers. 

Sood et al. examined ABS samples for their compressive strength. They conclude that for high compressive strength, the connection of the individual material webs to each other must have a high strength. To achieve this, the distortion during printing must be minimized. Furthermore, anisotropic effects must be minimized. These are caused by the alignment of the polymer molecules to each other when leaving the nozzle or through pores in the direction of the extrusion [25]. 

Abbas et al. investigated the compressive strength as a function of infill density on polylactic acid (PLA) samples. Only one type of infill structure, straight-line infill, was investigated. In straight-line infill, material webs are parallel with an air gap between them. With each layer change, the pattern is rotated 90° so that a kind of grid pattern is created when viewed from above. Abbas et al. showed that the compressive strength of the test specimens increases with increasing infill density. The test specimens with 80% infill showed the highest compressive strength of 30 MPa. Specimens with 100% infill were not examined [26].

Dey et al. compared the data from a large number of research papers. Their work examined samples made of ABS, PLA, Ultem, PEEK, and PC, as well as blends of these materials. According to this, the compressive strength should increase with increasing layer height. The infill density, as well as the number of contours or the wall thickness, also have a positive influence on the compressive strength. The orientation of the components in the installation space significantly influences the compressive strength since the FFF process generally produces anisotropic components [27].

Bakthiari et al. investigated the effect of layer height, extrusion width, nozzle temperature, and printing speed on the compressive properties and surface smoothness of FFF parts made of PLA. The results indicated that lower layer heights lead to better properties in general. Extrusion width had the second biggest impact, after layer height, in the case of compressive strength and specific strength. The optimal design for both high compressive properties and surface smoothness were determined as a 0.05 mm layer height, 0.65 mm extrusion width, 205 °C nozzle temperature, and 70 mm/s print speed. The main failure mechanism observed by SEM analysis was delamination between layers, occurring at highly stressed points near the stitch line of the PLA prints. All samples were produced with 100% infill density and 0°/45° grid pattern [28].

In a separate study, Borah et al. conducted a statistical analysis to examine the impact of infill density, print speed, and infill pattern on surface roughness, hardness, tensile strength, and elongation. Their evaluation revealed that high infill density and lower printing speeds obtained the highest tensile strength [29]. 

Arikan et al. conducted compression tests on PLA samples with varying infill patterns and densities. They found that triangular and tri-hexagonal patterns exhibited superior strength at higher densities, although their analysis focused solely on strength in the print direction [30].

The material considered in this paper is Polyetheretherketone (PEEK), a member of the Polyaryletherketone (PAEK) family [31]. PEEK stands out as a semi-crystalline thermoplastic known for its exceptionally high melting point, reaching 343 °C, which sets it apart from other thermoplastics. Its remarkable mechanical properties, strong biocompatibility, and chemical resistance render PEEK suitable for a wide array of applications [32]. A large area of application is in medical technology. Here, PEEK is characterized by its biocompatibility and is increasingly used in the field of individually manufactured implants [33,34,35,36]. Manufacturers typically specify its tensile strength as 100 MPa and compressive strength as up to 125 MPa [37]. 

Rahman et al. investigated the tensile, compressive, flexural strength, and impact resistance of PEEK specimens produced using the FFF process. However, their analysis focused solely on solid printed samples. The tensile strength ranged from 50 MPa to 75 MPa, depending on the orientation of the solid infill grid. Compressive strength fell from 64 MPa to 85 MPa [32].

Wu et al. achieved a compressive strength of 60.9 MPa in their PEEK specimens tested for compressive strength. This value corresponds to 76.7% of the strength exhibited by injection-molded comparative specimens that withstood a compressive force of 118 MPa. The FFF-printed specimens were manufactured using a layer thickness of 0.3 mm [38].

In the research conducted by Wächter et al., efforts were made to correlate the strength values of PEEK samples with the welding strength of individual material layers. Their findings revealed that the connection of individual material layers strengthened with increasing nozzle temperature and build chamber temperature. The highest strength was achieved at the maximum investigated values of 450 °C nozzle temperature and 250 °C build chamber temperature [39].

Timoumi et al. explored the application of PEEK in implants. They found that the highest tensile strength of FFF PEEK samples, using a honeycomb infill, was achieved with a nozzle temperature of 450 °C, a layer thickness of 0.1 mm, and a print speed of 20 mm/s. Additionally, they demonstrated that increasing the infill density resulted in greater strength [40]. A summary of the research carried out is listed in Table 1 and shows the research gap in the field of compression behavior of PEEK.

The primary objective of this study is to assess infill strategies for Fused Filament Fabrication (FFF) printed PEEK and evaluate their suitability for rapid tooling applications. Given the need for cost-effective PEEK utilization in rapid tooling and the existing gaps in the properties of PEEK components, this research will address the following inquiries:

1. How does thermal post-treatment of PEEK affect its compression properties?
2. What impact does the choice of material have on the characteristics of the infill patterns?
3. How do isotropic and anisotropic infill patterns perform, and what significance does the orientation of the components within the build space hold?
4. How do physical parameters, such as infill density and layer thickness, influence the compression properties?
5. What infill pattern and density are required to achieve efficient and economical utilization in Rapid Prototyping Tooling (RPT) for PEEK molds?

## 2. Materials and Methods

**Figure 1:**
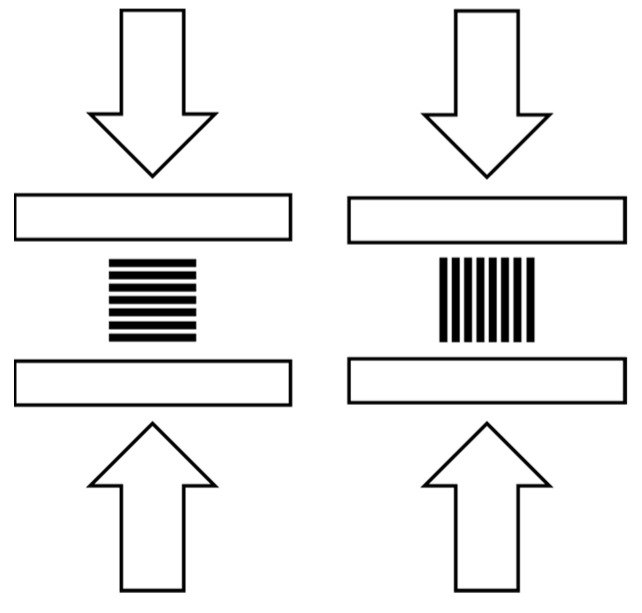
Uniaxial compression test: Z test direction (**left**) and XY test direction (**right**).

The test setup is divided into four sections. Starting with an investigation of the influence of thermal post-treatment of PEEK (3DXTECH—THERMAX PEEK; Grand Rapids, MI, USA) on compressive strength using solid PEEK samples (100% infill). In the second section, samples made of PEEK and PETG (DAS Filament, Emskirchen, Germany) are examined. Three anisotropic infill patterns, grid, triangle, and honeycomb, and two isotropic infill patterns, gyroid and cubic, are examined in a systematic parameter investigation. Two materials are used to exclude the influence of the material on the compressive strength. Using the best-performing infill strategies from the first section, samples with varying infill densities are manufactured in the third stage. 15%, 30%, and 45% samples provide a basis for predicting strength as a function of infill density. In the last test section, the layer thickness, which was kept constant in the previous series, is examined to determine its influence on the strength. All tests were carried out in compliance with DIN-604 [43]. For each test, six samples were manufactured and tested. Three in the Z direction and three in the XY direction, as shown in Figure 1. All steps are listed in Table 2. Finally, a vulcanization mold is made of PEEK and subjected to an injection molding test.

### 2.1. Specimen Design 

**Figure 2:**
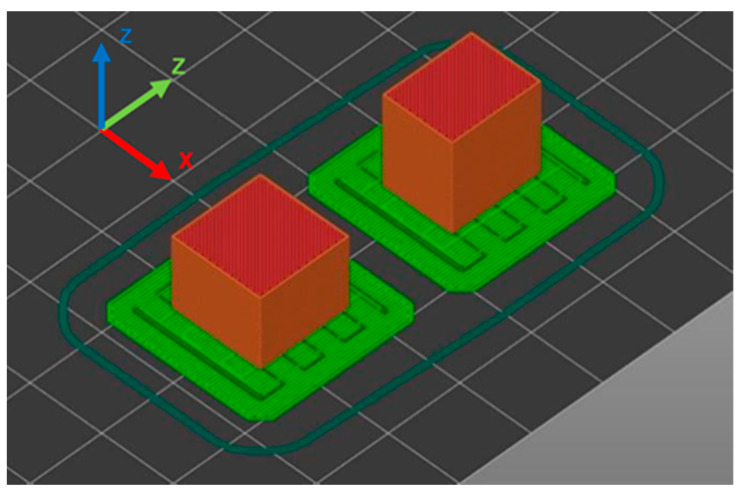
Sample orientation in slicer Z (**left**) and XY (**right**).

**Figure 3:**
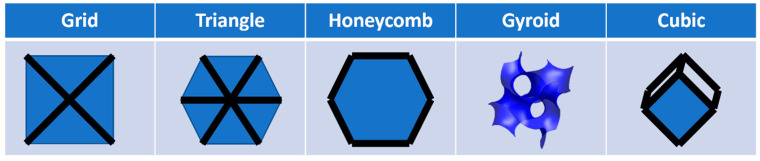
Different infill designs (Gyroid [44]).

The standard sample size for a compression test is a rectangular body with 10 × 10 × 4 mm³. However, depending on the infill density and pattern, the slicer will produce less than a complete repetition of the infill pattern, resulting in inhomogeneous filling and density distribution. The sample body was adapted to a 12.7 × 12.7 × 10 mm³ cube (Figure 2) so that all infill patterns can be formed entirely and the DIN-604 standard still applies [43]. All samples were printed with one perimeter to have equal and realistic test conditions on all specimens. The used infill designs are shown in Figure 3.

### 2.2. FFF Laboratory Plant

The laboratory printer used to manufacture the samples is equipped with an all-metal hot-end that can be heated to 500 °C and a heated glass bed that can be heated to 160 °C. The printer does not have an enclosure thus, parts were printed at room temperature. Previous tests have shown that the best results regarding porosity and toughness can be achieved with a nozzle temperature of 410 °C and a maximum temperature of 160 °C for the heated bed. Printing parameters that have not been changed during all tests are listed in the following Table 3.

Although the print speed is the same for all samples print duration can still vary. This is caused by the print head accelerating and decelerating on print moves. Patterns like honeycomb and gyroid with a lot of direction changes do not reach the full print speed and take longer to print. Print times for 10 samples are shown in Table 4. For 30% and 45% density print times for honeycomb and gyroid patterns are significantly longer than for grid, triangle, and cubic.

### 2.3. Uniaxial Compression Test 

The testing machine used was an Allround Line Z250 from ZwickRoell GmbH & Co. KG (Ulm, Germany). Specimens were preloaded with 20 N and compressed with 5 mm/min until breakage occurred or 30% deformation was reached. Stress and Strain were recorded. 

### 2.4. Thermal Post Treatment

A 3DGence (Przyszowice, Poland) laboratory furnace was employed to perform thermal post-treatment on the specimens. This thermal aging process is aimed at facilitating post-crystallization. The samples underwent a carefully controlled heating process, gradually reaching the desired temperature of 250 °C over an 11-h period. Subsequently, they were maintained at this temperature for a duration of 5 h after which they were slowly cooled back to room temperature over a span of 15 h. This gradual cooling was implemented to ensure even cooling and prevent the induction of stress that could result from quenching. The thermal post-treatment is based on the recommendations of the material manufacturers [45] and a specially conducted study [14].

### 2.5. Proof of Concept

With the best results from the preliminary tests, a PEEK mold is printed at room temperature. The layer thickness is 0.1 mm, the nozzle temperature is 410 °C, and a cubic infill with a density of 30% is used. The mold is placed in an aluminum master mold, heated to 200 °C, and then filled with an EPDM using a manual laboratory press. 

## 3. Results

### 3.1. Effect of the Post-Heat Treatment

**Figure 4:**
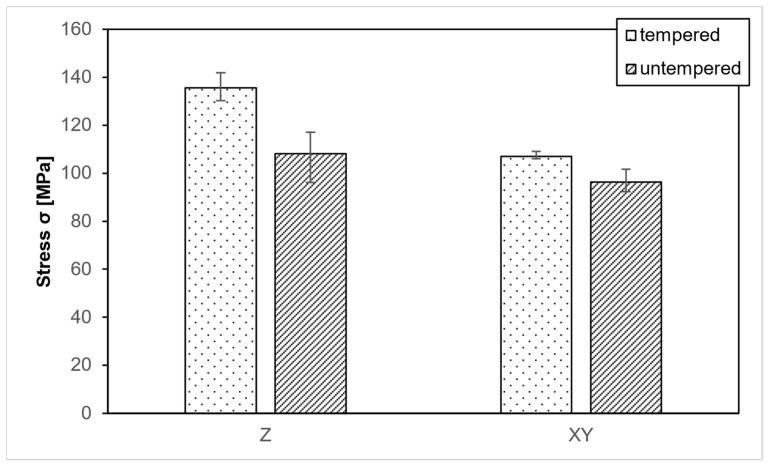
PEEK-Tempered and untempered samples with 100% infill.

PEEK samples were 3D printed with solid (100%) infill to verify the testing method. Some samples were examined without further treatment, while others underwent a heat treatment before testing. The treated samples achieved an average compressive strength of 135.6 MPa in the Z-direction and 107 MPa in the XY-direction (Figure 4). These values are higher than those reported in other research studies, likely due to the optimal parameters explored in these works, which were combined in this study [37].

Furthermore, the unique aspect of the strategy under examination involves the room-temperature Fused Filament Fabrication (FFF) production of PEEK components. This approach is geared towards creating amorphous components, which only undergo thermal post-processing as a secondary step. This methodology effectively mitigates the internal stresses that typically arise during FFF at elevated build chamber temperatures (BCT) when transitioning from an amorphous to a crystalline state. The primary objective is to enhance the bonding between individual layers by controlling the density shift.

The evaluated compressive strength is only slightly above the specified 125 MPa compressive strength for PEEK, which could be attributed to the use of material from a different manufacturer with a varying composition. Additionally, only the solid samples experience an increase in cross-sectional area due to compression, which is not captured by the measurement system. As a result, the actual stress in the component is slightly lower than the measured stress. Consequently, the values are realistic and fall within a very good range. Samples that did not undergo heat treatment achieved an average of 108 MPa in the Z-direction and 96.3 MPa in the XY-direction. Heat treatment led to a significant increase in strength in both directions. All subsequent samples are heat-treated before testing to achieve maximum strength.

### 3.2. Material Comparison

To explore different infill patterns, 30 samples for each material and three for each test series were manufactured using PEEK and PETG. The PEEK specimens were printed using a novel process strategy at room temperature and a nozzle temperature of 410 °C, while the PETG samples served as a reference to assess the impact of material and infill variations on strength.

**Figure 5:**
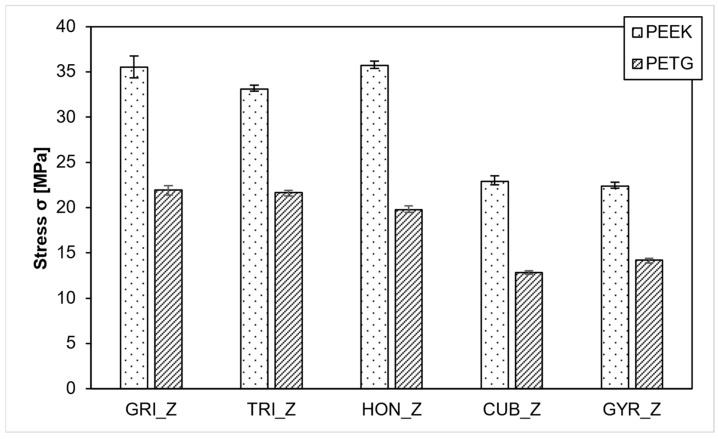
PEEK-Compressive strength in Z direction.

As depicted in Figure 5, the grid, triangle, and honeycomb infill patterns exhibited greater strength in the Z direction compared to the cubic and gyroid infill patterns. Both materials displayed higher tensile stress (σ) in the Z-direction for the anisotropic infill patterns. In the case of PEEK, the honeycomb infill slightly outperformed the grid infill with strengths of 35.7 MPa and 35.6 MPa, respectively. The triangle infill achieved a slightly lower strength, averaging 33.1 MPa. For PETG samples, the grid infill recorded 22 MPa, and the triangle infill recorded 21.7 MPa, both surpassing the honeycomb infill at 19.8 MPa. The isotropic infills, gyroid and cubic, displayed considerably lower values for both materials. In PEEK samples, the cubic infill led with 22.9 MPa, slightly ahead of the gyroid infill at 22.3 MPa. In PETG samples, the order was different, with the gyroid infill at 14.2 MPa and the cubic infill at 12.8 MPa. As expected, PEEK outperformed PETG, and both materials exhibited consistent behavior regarding the infill strategy employed.

**Figure 6:**
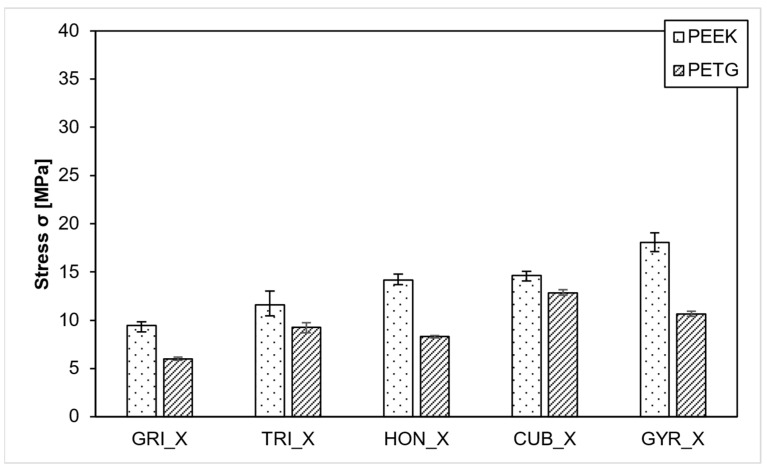
Compressive strength in XY direction.

In the XY direction, the isotropic infills achieved higher strengths than the anisotropic infills, as illustrated in Figure 6. The gyroid infill averaged 18 MPa for PEEK, surpassing the cubic infill at 14.6 MPa. Conversely, in the PETG samples, the cubic infill averaged 12.8 MPa, making it more stable than the gyroid infill at 10.6 MPa. The grid infill recorded the lowest values for both materials. Surprisingly, the PEEK grid samples, averaging 9.4 MPa, were outperformed by the PETG samples with gyroid and cubic infills despite PEEK being expected to be significantly stronger.

**Figure 7:**
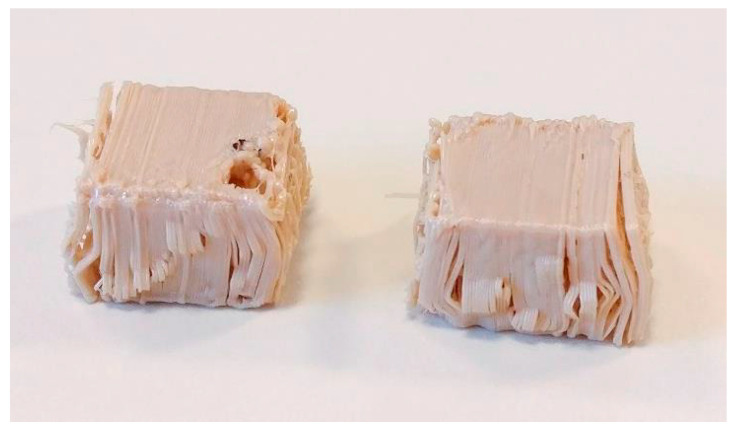
PEEK XY Sample after testing.

While the results for the z-direction are consistent, the minimal strength difference between PEEK and PETG in the XY direction, especially for cubic infill, warrants further investigation. One possible explanation for the low compressive strength of the PEEK samples lies in the examination of the test specimens post-strength analysis. These samples exhibited delaminations at every edge where the material had folded, as observed in Figure 7. The layers had separated from each other over short distances, and in some instances, entire layers had detached. The PETG specimens did not show this behaviour and buckled at the edges rather than separate layers. This behavior suggests significantly reduced layer adhesion for PEEK, which is unexpected given that state-of-the-art parameters were used for sample production. Previous tests had indicated that these parameters were superior to others. This suggests underlying issues within the process, such as low-layer adhesion, which require further investigation. According to Wächter et al., higher build chamber temperatures lead to better adhesion of layers. We could not reproduce these results and will investigate further.

### 3.3. Effect of the Infill Density

As the next step, two infill patterns, the anisotropic grid pattern, and the isotropic cubic pattern were printed with a density of 15%, 30%, and 45% and tested.

The grid pattern was chosen for its outstanding performance with PEEK and PETG in the prior test. Also, a grid can be printed much faster than a honeycomb, which is an economical advantage considering the manufacturing costs of molds. The cubic infill was chosen because it can also be printed faster than gyroid and has better strength when printed in PETG. 36 Samples in total were tested.

**Figure 8:**
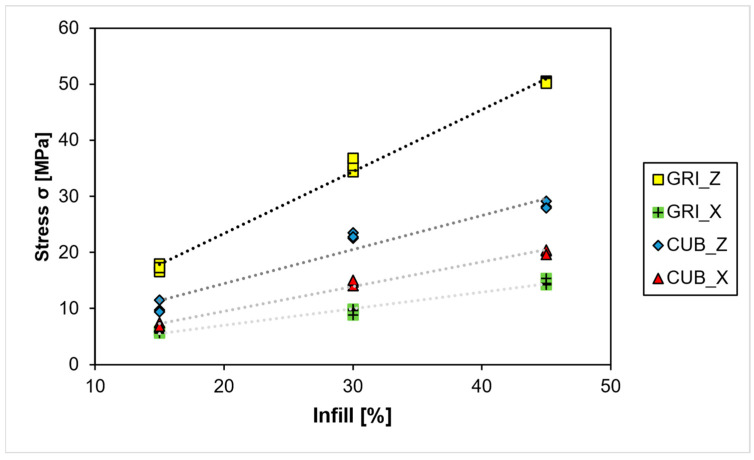
PEEK–Trendlines for different infill densities.

Although minor deviations at 30% can be seen with the grid pattern, a clear linear correlation between infill density and compressive stress can be seen (Figure 8). The anisotropy of the grid pattern can be seen by the difference in line pitch and between Z and XY. Cubic infill lines have nearly identical pitches and less difference between Z and XY, which the much higher isotropy can explain.

Infill strength can be predetermined for the grid pattern with the following Equation (1):

(1) $$\sigma \left(Z\right)=4.818+1.0688\;*Density\;[\%]\;(R^{2}=99.59\%)\;\sigma \left(XY\right)=2.097+0.2853\;*Density\;[\%]\;(R^{2}=97.28\%)$$

Infill strength can be predetermined for the cubic pattern with the following Equation (2):

(2) $$\sigma \left(Z\right)=3.26+0.5629\;*Density\;[\%]\;(R^{2}=92.67\%)\;\sigma \left(XY\right)=1.378+0.4088\;*Density\;[\%]\;(R^{2}=97.83\%)$$

It can be expected that these equations describe the actual behavior of infill roughly from 10% to 70%. As Luzanin et al. described, outside of this range, the influence of perimeters and other factors will have a much higher impact on the strength than the infill pattern [24].

### 3.4. Effect of the Layer Height

In the final phase of the study, the impact of different layer heights on compressive strength, involving 36 specimens with layer thicknesses of 0.1 mm, 0.15 mm, and 0.2 mm were examined.

**Figure 9:**
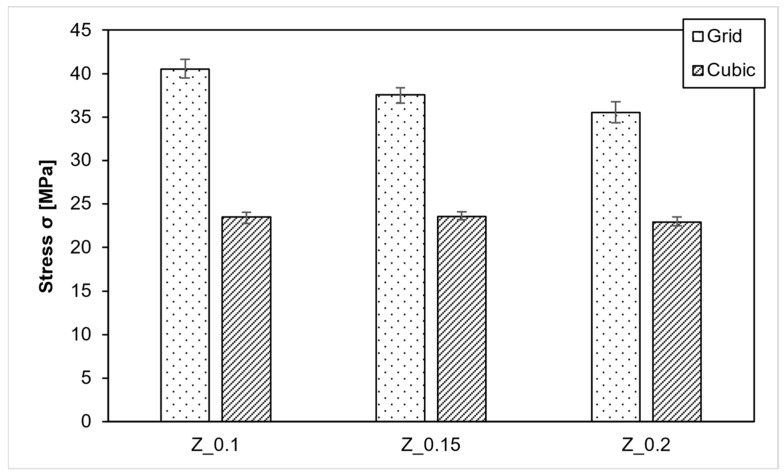
PEEK-Layer height variation Z direction.

The results in the Z-direction, as presented in Figure 9, reveal that compressive strength increases as the layer thickness decreases. The average compressive strength for grid infill at 0.15 mm is 37.6 MPa, approximately 2 MPa higher than at 0.2 mm. Further reducing the layer thickness to 0.1 mm results in a compressive strength of 40.5 MPa, approximately 3 MPa higher than 0.15 mm.

Cubic infill, on the other hand, exhibits minimal differences between 0.1 mm and 0.15 mm layers. With 0.1 mm layers, the average compressive strength reaches 23.52 MPa, while with 0.15 mm layers, it is 23.54 MPa. Both values surpass the 22.9 MPa achieved with 0.2 mm layers.

**Figure 10:**
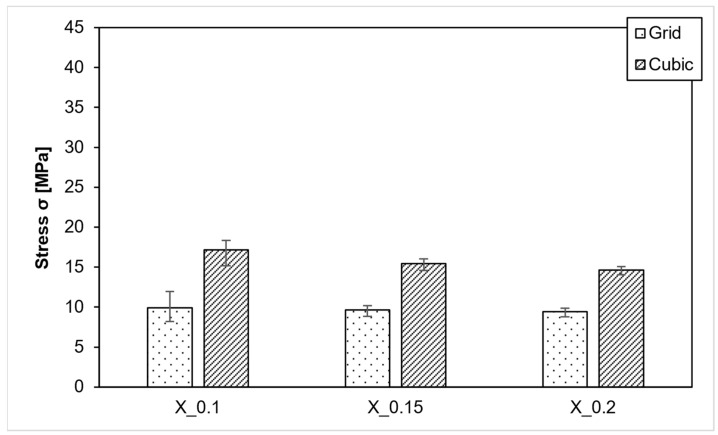
PEEK-Layer height variation XY direction.

In the XY direction, grid infill shows nearly no differences, as demonstrated in Figure 10. At a layer thickness of 0.2 mm, the strength is 9.4 MPa; at 0.15 mm, it is 9.6 MPa; and at 0.1 mm, it is 9.9 MPa. The samples with a layer thickness of 0.1 mm exhibit slightly more variation, ranging from 8.2 MPa to 11 MPa, than the other values.

For cubic infill, the values display more variability as layer thickness decreases. On average, models are more stable with thinner layers. With 0.1 mm layers, a strength of 17.2 MPa is achieved, while with 0.15 mm layers, it is 15.5 MPa, and with 0.2 mm layers, it is 14.6 MPa.

Since the strength either increases or remains consistent as layer thickness decreases for both infill patterns, there are no concerns regarding the previously mentioned equations. Vulcanization tools designed using these equations can achieve the specified strength even with layer thicknesses of 0.15 mm or 0.1 mm, and in some cases, they might even offer increased stability.

**Figure 11:**
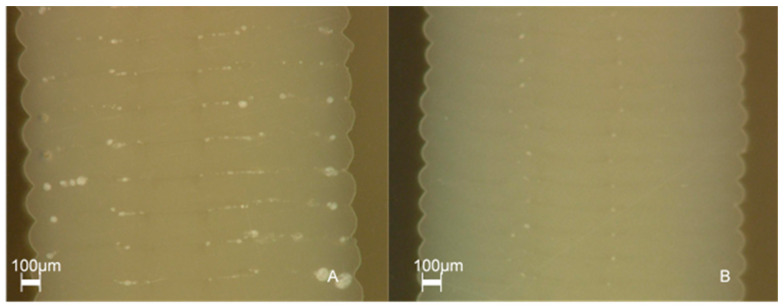
Micrographs of different layer heights printed at 410 °C: (**A**) 0.15 mm; (**B**) 0.1 mm.

In a supplementary study, the interlayer bonding at different layer thicknesses was evaluated using micrographs. The following Figure 11 shows the micrographs of test specimens with a layer thickness of 0.15 mm and 0.1 mm. The significant reduction of the pores by minimizing the layer thickness to 0.1 mm is visible.

**Figure 12:**
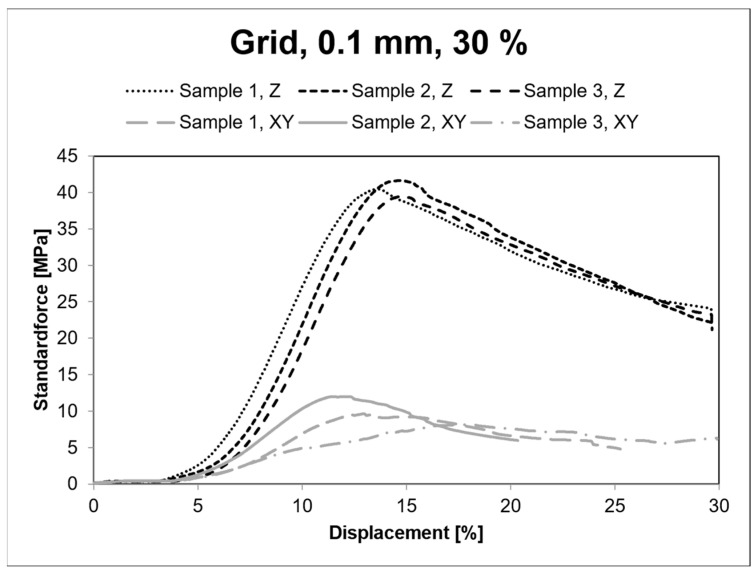
Stress-strain diagram for 0.1 mm layer height.

Figure 12 shows the stress-strain diagram for grid infill with 30% density and 0.1 mm layer height.

### 3.5. Proof of Concept

**Figure 13:**
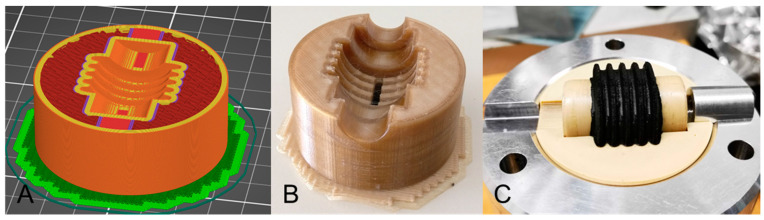
(**A**) Sliced PEEK mold with cubic infill; (**B**) Amorphous PEEK mold; (**C**) Master and PEEK mold and rubber component after molding.

Figure 13 shows the infill structure, the amorphously solidified mold, and the mold after the vulcanization process with a rubber component. With a closing force of 100 kN on a mold surface of 5000 mm², it was anticipated that this would represent the maximum force. As a result, the calculated infill density for cubic infill is 29.74%. The proof of concept validates the successful utilization of PEEK with a minimal infill density of 30%. The rubber part has the typical FFF surface pattern that is created by the stair-step effect. Except for the cast skins, the rubber part is completely molded. The mold and the mold core have not suffered any defects despite the high temperatures and the high injection pressure. Even after an injection molding series of more than 50, no defects have been detected on the mold.

## 4. Discussion and Conclusions

In addition to the classic areas of application in the automotive and medical technology sectors [46], PEEK is a pioneering material for rapid tooling. The tests conducted so far show promising approaches for use in vulcanization tools. In addition to prototyping, tools can also be qualified for series production. The complex processing of PEEK, due to the narrow process window, should not be neglected. Using PEEK in rapid tooling enables companies to produce tools and the resulting injection-molded components economically and on time. Even though PEEK is very expensive, costing 600–800 €/kg, tool production using aluminum or tool steel is significantly higher. A comparison shows that the PEEK mold investigated here is 72% cheaper than conventional mold production using tool steel and aluminum. In particular, the infill strategy contributes to a resource-efficient design of the molds and saves costs and time. At the same time, the ratio of material input and mechanical properties is maintained.

First, the results obtained in this research have shown that PEEK can be printed at room temperature and has extraordinary mechanical properties, reaching higher values than (84.49 MPa [32]; 60.9 MPa [38]). This contrasts with the classical FFF strategies of PEEK, which relied on a high build room temperature above the glass transition temperature [47,48,49,50]. The samples fabricated by this strategy are amorphous and require thermal post-treatment. As previously timed in a study conducted by the authors, annealing increases the degree of crystallinity (DOC) [14]. This leads to improved morphology and eventually to improved mechanical properties. The annealing process gives the molecules sufficient time to rearrange themselves, which leads to the recommendation to subject PEEK components to thermal post-treatment. The mechanical properties are increased by up to 20% in Z and up to 10% in XY.

Different infill densities were tested for grid and cubic to obtain a relationship between compressive strength and infill density. As multiple others found the strength rises with higher infill densities. The established regression equations can be used to estimate part strength [21,22,26,27].

Investigating the interplay between the layer thickness and the compressive strength has shown that the compressive strength can be increased by reducing the layer thickness. This can be attributed, among other things, to an improved print image. The reduction of the layer thickness leads to a decrease in inter-layer pores and, consequently, to an optimization of the inter-layer bonding. This has a positive effect on neck growth and can be seen in the micrographs. Pores, however, reduce the area over which force is absorbed and distributed. This leads to local overstress. The occurrence of layer delamination does not necessarily imply a flaw in the print quality. It is essential to consider that the staircase effect inherently introduces a notch effect, which can have a detrimental impact even when interlayer bonding is flawless. Additionally, it is important to remember that the shell constitutes only one wall thickness, and thus, the evaluation of the infill strategy cannot solely rely on the external appearance of the test specimen. The micrographs that were also obtained concurrently illustrate the enhanced interlayer bonding among adjacent layers. 

Sood et al. [25] and Li et al. [51] also observed similar results in their investigations using other materials. However, Dey et al. assume in their study that the increase in layer thickness leads to an increase in compressive strength; this assumption cannot be confirmed in the context of this work [27].

The findings presented in this study serve as a foundational platform for future research in the realm of infill strategies and the characterization of PEEK. Initially, it is imperative to conduct assessments concerning additional directional properties related to component placement within the build space. Moreover, several influential factors, including the impact of wall thickness or surface roughness, were not considered in this study. Given the thermal stresses to which a vulcanization mold is exposed, it is advisable to incorporate investigations involving cyclic thermal loads.

In summary, the following conclusions and contributions for the future can be drawn from the work:

1. Processing of PEEK at room temperature is possible, but it should be noted that the components must be thermally post-treated to exploit the full potential of the mechanical properties.
2. For components subject to significant stress in the Z direction, the most effective infill pattern is the grid, offering a strength of 35.6 MPa when using a 30% density. Conversely, when dealing with high-stress scenarios in all directions, it is recommended to employ cubic infill, which achieves a strength of 14.6 MPa at a 30% infill density.
3. A layer height of 0.1 mm results in greater strength compared to a 0.15 mm layer height, which, in turn, exhibits greater strength than a 0.2 mm layer height. This effect is more pronounced with cubic infill, where transitioning from a 0.2 mm to a 0.1 mm layer height increases strength by 15%, whereas the same layer height change with a grid pattern only provides a 5% strength gain. Interlayer bonding plays an essential role in influencing compressive strength.
4. Using the adapted infill rate, resources and costs can be saved in the rapid tooling of vulcanization molds. Production costs can be reduced by more than 70%. PEEK is suitable for the vulcanization injection molding of rubber components thanks to its excellent material properties.

The findings yield significant insights for both practitioners of Fused Filament Fabrication (FFF) and users of rapid tooling. Firstly, this research demonstrates the potential for optimizing the design of vulcanization tools with greater efficiency. It underscores the substantial impact of infill strategies and the untapped possibilities they offer. In an era characterized by short product life cycles, the manufacturing strategies presented in this study enable more agile and adaptable responses, all while maintaining economic viability. Notably, the utilization of PEEK in rapid tooling expedites prototype development and metal mold creation, while also revealing potential applicability in small-scale production runs. It is important to acknowledge the effects of polymer aging when employing polymers. Although prolonged utilization of PEEK in vulcanization is not entirely precluded, it is subject to limitations. Extended exposure, as demonstrated by the authors by long-term tests exceeding 720 h at 200 °C, results in a gradual decline in mechanical properties [14].

## Figures and Tables

**Table 1:** Research overview in the field of FFF.

Study	Analysis	Material	Parameter	Results
Pandzic [21]	Tensile	PLA	Pattern (Grid, Lines, Triangles, Tri-Hexagonal, Cubic, Cubic-Subdivision, Octet, Quarter Cubic, Concentric, Zig Zag, Cross, Cross 3D, Gyroid)	Concentric Infill has the highest tensile strength
Cerda [22]	Flexural	PLA	Density (20%, 40%, 80%, 100%), Orientation (X, Y, Z)	Higher infill higher strength Orientation is a key parameter
Mishra [23]	Flexural	ABS	Contours (1, 3, 5), layer thickness (0.18 mm, 0.25 mm, 0.33 mm), raster width, raster angle, part orientation, air gap	Higher strength with thicker walls
Luzanin [24]	Flexural	PLA	Layer height (0.1 mm, 0.2 mm, 0.3 mm), orientation (0°, 30°, 60°), density (10%, 20%, 30%)	Layer thickness has the greatest effect on strength The combination of orientation and density also has a significant influence
Sood [25]	Compression	ABS	Layer thickness, orientation, raster angle, raster width, air gap	Good connection between material webs is important for strength
Abbas [26]	Compression	PLA	Straight-line infill, 20%, 35%, 50%, 65%, 80%	Higher density leads to higher strength
Bakthiari [28]	Compression, Surface roughness, Density	PLA	layer height (0.05, 0.15, 0.25), extrusion width (0.45 mm, 0.55 mm, 0.65 mm), nozzle temperature (190 °C, 205 °C, 220 °C), printing speed (30 mm/s, 50 mm/s, 70 mm/s)	0.05 mm layers and 0.65 mm extrusion width
Borah [29]	Surface roughness, Hardness, Tensile, Elongation	PEEK	infill density (60%, 70%, 80%), print speed (25 mm/s, 30 mm/s, 35 mm/s), and infill pattern (octet, gyroid, triangular)	High density, low print speed
Arikan [30]	Compression	PLA	Pattern (Line, Cubic, Octet, Triangles, Tri-Hexagon), density (10%, 15%, 20%)	Triangular and tri-hexagonal, high density
Rahman [32]	Tensile, Compression, Flexural, Impact	PEEK	Solid infill angle (0°, 90°, 0°/90°)	Compression: 50–75 MPa
Wu [38]	Compression	PEEK	FFF compared to Injection molding	76.7% of injection-molded force
Wächter [39]	Tensile	PEEK	Nozzle (430 °C, 440 °C, 450 °C) and build chamber temperature (150 °C, 200 °C, 250 °C)	Best results: Nozzle 450 °C, Chamber 250 °C
Paszkiewicz [41]	DSC TGA Tensile Impact XRD	PEEK/PEKK	Nozzle temperature (360–380 °C), print speed (15–25mm/s), infill (20–100%), layer height (0.2 mm), BCT (90 °C)	PEKK HT with the most promising mech. properties and less bacterial adhesion for medical use
Timoumi [40]	Tensile	PEEK	Nozzle temperature (420 °C, 450 °C), print speed (20 mm/s, 30 mm/s), layer thickness (0.1 mm, 0.2 mm), density (40%, 55%, 70%)	Nozzle 450 °C, 0.1 mm layers, 20 mm/s speed Higher density leads to higher strength
Mrowka [42]	Tensile Tree-point-bending Impact	PEEK	Nozzle temperature (425 °C), print speed (30 mm/s), infill (100%), layer height (0.15 mm), orientation (0°, 90° X, 90° Y) Parts were tested in amorphous and crystalline form	Lowest mech. properties of vertical specimen, 4.82–43.67 MPa tensile strength, impact strength 6.57–112.09 kj/m²

**Table 2:** Experimental setup.

Section	Samples	Parameter
1	12—3 per point	Density: 100% Heat treatment: Y/N Orientation: XY/Z Material: PEEK
2	60—3 per point	Density: 30% Pattern: Grid, triangle, honeycomb, cubic, gyroid Orientation: XY/Z Material: PEEK/PETG
3	36—3 per point	Density: 15%, 30%, 45% Pattern: Grid, cubic Orientation: XY/Z Material: PEEK
4	36—3 per point	Density: 30% Pattern: Grid, cubic Layer thickness: 0.1 mm, 0.15 mm, 0.2 mm Orientation: XY/Z Material: PEEK

**Table 3:** Printing parameters.

Parameter	Value
Nozzle temperature	410 °C
Build plate temperature	150 °C
Chamber temperature	Room temperature
Print speed	40 mm/s

**Table 4:** Print duration for ten samples as calculated by the slicer.

Pattern/Density	Grid	Triangle	Honeycomb	Cubic	Gyroid
15%	2 h 2 min	1 h 50 min	2 h 16 min	1 h 58 min	2 h 20 min
30%	2 h 18 min	2 h 23 min	3 h 22 min	2 h 25 min	3 h 30 min
45%	2 h 56 min	2 h 55 min	4 h 43 min	2 h 53 min	4 h 50 min

## Data Availability

Data are contained within the article.
